# *Isospora atticorae* n. sp. (Apicomplexa: Eimeriidae) from the white-thighed swallow *Atticora tibialis* (Cassin, 1853) (Passeriformes: Passeri: Hirundinidae) in Brazil

**DOI:** 10.1007/s11686-026-01371-5

**Published:** 2026-08-10

**Authors:** Leandro Dorna-Santos, Carlos Nei Ortúzar-Ferreira, Mariana de Souza Oliveira, Alan Fecchio, Viviane Moreira de Lima, Bruno Pereira Berto

**Affiliations:** 1 Programa de Pós-graduação em Biologia Animal, Instituto de Ciências Biológicas e da Saúde, Universidade Federal Rural do Rio de Janeiro, Seropédica, Rio de Janeiro, Brasil; 2 Departamento de Patologia, Reprodução e Saúde Única, Faculdade de Ciências Agrárias e Veterinárias, Universidade Estadual Paulista Júlio de Mesquita Filho, Jaboticabal, São Paulo, Brasil; 3 Departamento de Biologia Animal, Instituto de Ciências Biológicas e da Saúde, Universidade Federal Rural do Rio de Janeiro, BR 465, Km 7, Seropédica, Rio de Janeiro, CEP 23890-000 Brasil

**Keywords:** Oocysts, Coccidia, Integrative taxonomy, Phylogeny, Atlantic forest

## Abstract

**Background:**

The diversity of coccidians in Neotropical wild avifauna remains significantly underestimated, particularly within the family Hirundinidae. While cosmopolitan and widely distributed hosts like *Hirundo rustica* Linnaeus, 1758 dominate the literature with three described *Isospora* spp., ecological traits of other swallows may drive different parasitic dynamics. This study provides the first integrative characterization of a coccidian parasite from *Atticora tibialis* (Cassin, 1853), a resident swallow strictly associated with complex and preserved Neotropical forest biomes.

**Methods:**

Fecal samples were collected from three White-Thighed Swallows, *A. tibialis*, captured using mist nets in the Itatiaia National Park, Southeastern Brazil, during field expeditions in 2023 and 2024. Oocysts were processed through standard flotation and morphological analyses. Molecular characterization was performed by extracting genomic DNA from isolated oocysts and sequencing of a locus of the mitochondrial cytochrome c oxidase subunit 1 (cox1) gene. Phylogenetic trees were reconstructed using Bayesian Inference and Maximum Likelihood.

**Results:**

One bird was positive for coccidia (33.3% prevalence), and a new species is formally proposed herein: *Isospora atticorae* Dorna-Santos et Berto n. sp. The sporulated oocysts are pyriform, measuring on average 24 × 21 μm. A unique, distinguishing feature among congeners parasitizing the same host family is the presence of a distinct micropyle. A single, robust polar granule is present, and an oocyst residuum is absent. Sporocysts are ellipsoidal to lemon-shaped, with a mean size of 16 × 10 μm. Stieda and sub-Stieda bodies are present. A sporocyst residuum occurs among the vermiform sporozoites, which exhibit refractile bodies and a barely discernible nucleus. The obtained cox1 gene sequences were identical and shared 97–99% similarity with *Isospora* spp. from oscine birds (Passeri). In the phylogenetic tree, *I. atticorae* n. sp. clustered within a well-supported monophyletic clade of oscine-associated *Isospora* spp., closely grouped with *Isospora* spp. from *Serinus canaria* (Linnaeus, 1758) and *Turdus migratorius* Linnaeus, 1766.

**Conclusions:**

The characterization of *I. atticorae* n. sp. highlights how the restricted distribution and forest dependence of *A. tibialis* favor localized parasite diversification and narrow host specificity within Neotropical biomes, contrasting with the dynamics of widely distributed migratory swallows. Globally, *I. atticorae* n. sp. is established as the first representative from the family Hirundinidae to be characterized from an integrative perspective through the sequencing of a mitochondrial locus, as well as the fifth nominally described species in this avian family, and the first in the Neotropical region.

## Introduction

The Atlantic Forest is recognized as a global biodiversity hotspot, characterized by high levels of endemism and significant environmental pressure. Within this biome, the Itatiaia National Park (Parque Nacional de Itatiaia – PNI) represents a crucial conservation unit; established in 1937 as Brazil’s first national park, it currently encompasses 11,943 hectares of protected area [1]. Situated in Southeastern Brazil, the PNI harbors a remarkable avian diversity, with an estimated richness of approximately 370 bird species [2, 3].

Wild avifauna serves as host to a vast diversity of parasites, among which coccidians represent one of the most biodiverse and ubiquitous groups [4–6]. These organisms are parasitic apicomplexans and are the etiological agents of coccidiosis, a disease of significant clinical and ecological impact [4, 7–9].

The order Passeriformes exhibits the highest diversity among the class Aves, comprising a vast array of families and species [10–11]. Within this order, the coccidian genus *Isospora* Schneider, 1881, stands out as the most prevalent. Morphologically, *Isospora* spp. are characterized by disporocystic, tetrazoic oocysts typically possessing Stieda bodies, and are thus well-established members of the family Eimeriidae [5, 12–13].

The passeriform family Hirundinidae Rafinesque, 1815, includes the white-thighed swallow *Atticora tibialis* (Cassin, 1853), a species with significant forest dependence typically associated with riverine habitats, waterfalls, and dense forest canopies [11, 14]. Despite the wide distribution of hirundinids, their coccidian parasites remain poorly understood. In this context, the present study provides a morphological description and molecular characterization based on the mitochondrial cytochrome c oxidase subunit I (cox1) gene of a new species of *Isospora* recovered from a white-thighed swallow *A. tibialis* captured in PNI.

## Materials and methods

### Sample collection

Two fieldwork expeditions were conducted in September 2023 and November 2024 at the PNI. Sampling was performed specifically at the initial kilometer of the Ruy Braga Trail (22°26’08.11"S; 44°37’27.07"W). A total of three adult white-thighed swallows, *A. tibialis*, of undetermined sex, were captured using mist nets. The birds were identified to the species level following established ornithological criteria [15–16] and photographed. Following capture, each individual was briefly maintained in an isolated box lined with clean paper to facilitate the collection of fecal samples. Once defecation occurred, the birds were released at the capture site. Fresh fecal droplets were individually transferred to centrifuge tubes containing a 2.5% potassium dichromate (K_2_Cr_2_O_7_) solution [17] to ensure oocyst preservation and sporulation.

## Morphological analyses

The fecal samples were processed and analyzed at the Laboratory of Biology of Coccidians (Laboratório de Biologia de Coccídios – LABICOC), Federal Rural University of Rio de Janeiro (Universidade Federal Rural do Rio de Janeiro – UFRRJ). All samples were incubated at room temperature (approximately 25 °C) for seven days to allow for oocyst sporulation. Oocysts were recovered by flotation in Sheather’s saturated sugar solution (specific gravity: 1.20) [18]. Coccidian prevalence was calculated as the ratio of positive hosts to the total number of individuals examined [19], while density was defined as the number of oocysts per fecal droplet [17, 19]. Morphological observations and morphometric analyses were conducted following the guidelines of Duszynski & Wilber [18] and Berto et al. [5]. Samples were examined using an Olympus BX binocular microscope (Olympus Optical, Tokyo, Japan) equipped with a Eurekam 5.0 digital camera (BEL Photonics, Monza, Italy). Line drawings, photomicrographs, and final plates were prepared and edited using CorelDRAW and Corel PHOTO-PAINT (CorelDRAW Graphics Suite 2020, Corel Corporation, Ottawa, Canada). All measurements are expressed in micrometres (µm) and are presented as ranges followed by the mean in parentheses.

## Molecular analyses

Following morphological characterization and photomicrography, six and seven oocysts were respectively isolated from two distinct fecal droplets on microscope slides. These oocysts were then transferred into microtubes containing 90 µL of phosphate-buffered saline (PBS), according to the protocol described by Dolnik et al. [20]. To ensure the disruption of the oocyst wall and complete lysis, the samples were subjected to four freeze–thaw cycles (alternating between − 4 °C and 96 °C) for 5 min each. Subsequently, 10 µL of proteinase K was added, and the suspension was incubated at 56 °C for 2 h. Genomic DNA (gDNA) extraction was performed using the Quick-DNA Kit (Zymo Research, Irvine, CA, USA) according to the manufacturer’s protocol. Polymerase chain reaction (PCR) was employed to amplify a specific locus of the mitochondrial cox1 gene (COIBF1, positioned at ~ 2250–2500 nt in the Eimeriidae mitochondrial genome) [21]. Each 25 µL reaction volume contained 3 µL of template gDNA (< 1 µg), 12.5 µL of GoTaq^®^ G2 Hot Start Colorless Master Mix (Promega, Madison, WI, USA) (1×), 0.25 µL of each primer (0.2 µM), and 9 µL of nuclease-free water. The amplification was conducted following the thermal cycling conditions established by Ortúzar-Ferreira et al. [21].

## DNA sequencing and phylogenetic analyses

Amplicons were sequenced in both directions using the original PCR primers by Ludwig Biotechnology (Alvorada, RS, Brazil) on an ABI Prism 3500 Genetic Analyzer (Applied Biosystems, Foster City, CA, USA) via the Sanger method. The resulting chromatograms were inspected and edited using Chromas v.2.6 (Technelysium Pty Ltd). Sequences were compared with those of related *Isospora* spp. available in the GenBank database using the Basic Local Alignment Search Tool (BLAST). Multiple sequence alignments were performed in MEGA v.10.2.6 using the ClustalW algorithm. *Eimeria tenella* (Railliet et Lucet, 1891) was selected as the outgroup. Phylogenetic relationships were reconstructed using both Bayesian Inference (BI) implemented in MrBayes v.3.2.7 [22] and Maximum Likelihood (ML) implemented in MEGA [23]. The best-fitting evolutionary models for each method were identified using the Model Selection tool in MEGA. BI was conducted under the GTR + G model for 1,000,000 generations, with trees sampled every 100 generations; the first 25% of trees were discarded as burn-in. ML analysis was performed under the TN93 + G model, and node support was assessed via 1,000 bootstrap replicates. The final phylogenetic trees were visualized and exported using FigTree v.1.4.4.

## Results

### Prevalence and description

Among the three specimens of *A. tibialis* captured along the Ruy Braga Trail in the PNI, one was positive for coccidian oocysts, resulting in a prevalence of 33%. Based on morphological features, the oocysts were assigned to the genus *Isospora*. However, the oocysts did not match the taxonomic characteristics of any *Isospora* sp. previously described from hosts within the family Hirundinidae (Table 1). Consequently, the material is described herein as a new species.

**Table 1:** Comparative morphology of nominal *Isospora* spp. recorded from Hirundinidae

Coccidia	Host	Locality	Reference	Oocysts								Sporocysts						
				Shape	Length (µm)	Width (µm)	L/W ratio	Wall (µm)	Micropyle (µm)	Polar granule (µm)	Residuum	Shape	Length (µm)	Width (µm)	L/W ratio	Stieda Body (µm)	Substieda body (µm)	Residuum (µm)
*Isospora hirundinis* Schwalbach, 1959	*Hirundo rustica* Linnaeus, 1758	Germany	Schwalbach [24],	ovoidal to ellipsoidal	24–34 (28.0)	20–29 (22.6)	(1.2)	(1.0)	absent	present	absent	piriform	(19)	(10.8)	(1.8)	present	present	present
*Isospora petrochelidon* Stabler et Kitzmiller, 1972	*Petrochelidon pyrrhonota* Vieillot, 1817	USA	Stabler & Kitzmiller [27]	ovoidal to ellipsoidal	23–30 (25.2)	19–25 (22.2)	not reported	smooth, (1.0)	absent	present, 2 to 3, flattened oval or discs, (4.0)	absent	lemon-shaped	16–22 (18.4)	10–12 (10.8)	not reported	prominent cap, (2.5 wide)	elipsoidal, (4 × 2.5)	compact granular mass, 6–9
*Isospora mehlhornii* Abd-Al-Aal, Ramadan & Al-Hoot, 2000	*H. rustica*	Egypt	Abd-Al-Aal et al. [28]	subspherical	26–32 (28.3)	26–32 (28.3)	not reported	(1.5)	absent	absent	absent	lemon-shaped	20–25 (23.0)	12–18 (14.4)	not reported	prominent, (1.5 × 2)	present	spherical
*Isospora zimmermani* McAllister et Hnida, 2023	*H. rustica*	USA	McAllister & Hnida [29]	subspheroidal to ovoidal	21–25 (22.8)	19–22 (20.8)	1.0–1.3 (1.1)	smooth, 1.0–1.4 (1.2)	absent	present, usually 1 (typically) to 3	absent	ovoidal to ellipsoidal	14–17 (15.2)	8–11 (9.6)	1.4–1.8 (1.6)	knob-like, 1.0–1.4 × 1.7–2.1 (1.3 × 1.9)	rounded, 1.7–2.2 × 2.9–3.5 (2.0 × 3.2)	compact sphere or dense irregular mass
*Isospora atticorae* Dorna-Santos et Berto n. sp.	*Atticora tibialis* (Cassin, 1853)	Brazil	present study	pyriform	21–27 (24.4)	20–23 (21.2)	1.0–1.3 (1.2)	smooth, 0.8–1.4 (1.1)	present, 2.0–4.2 (2.9)	present, single and robust, 2.5–3.9 × 1.4–3.2 (3.3 × 2.2)	absent	ellipsoidal to lemon-shaped	14–18 (16.1)	9–11 (10.3)	1.4–1.7 (1.6)	knob-like, 0.7–1.4 × 1.4–1.9 (1.1 × 1.7)	rounded to trapezoidal, 1.5–2.5 × 2.6–3.7 (2.0 × 3.3)	granules totally scattered or loosely arranged

**Fig. 1:**
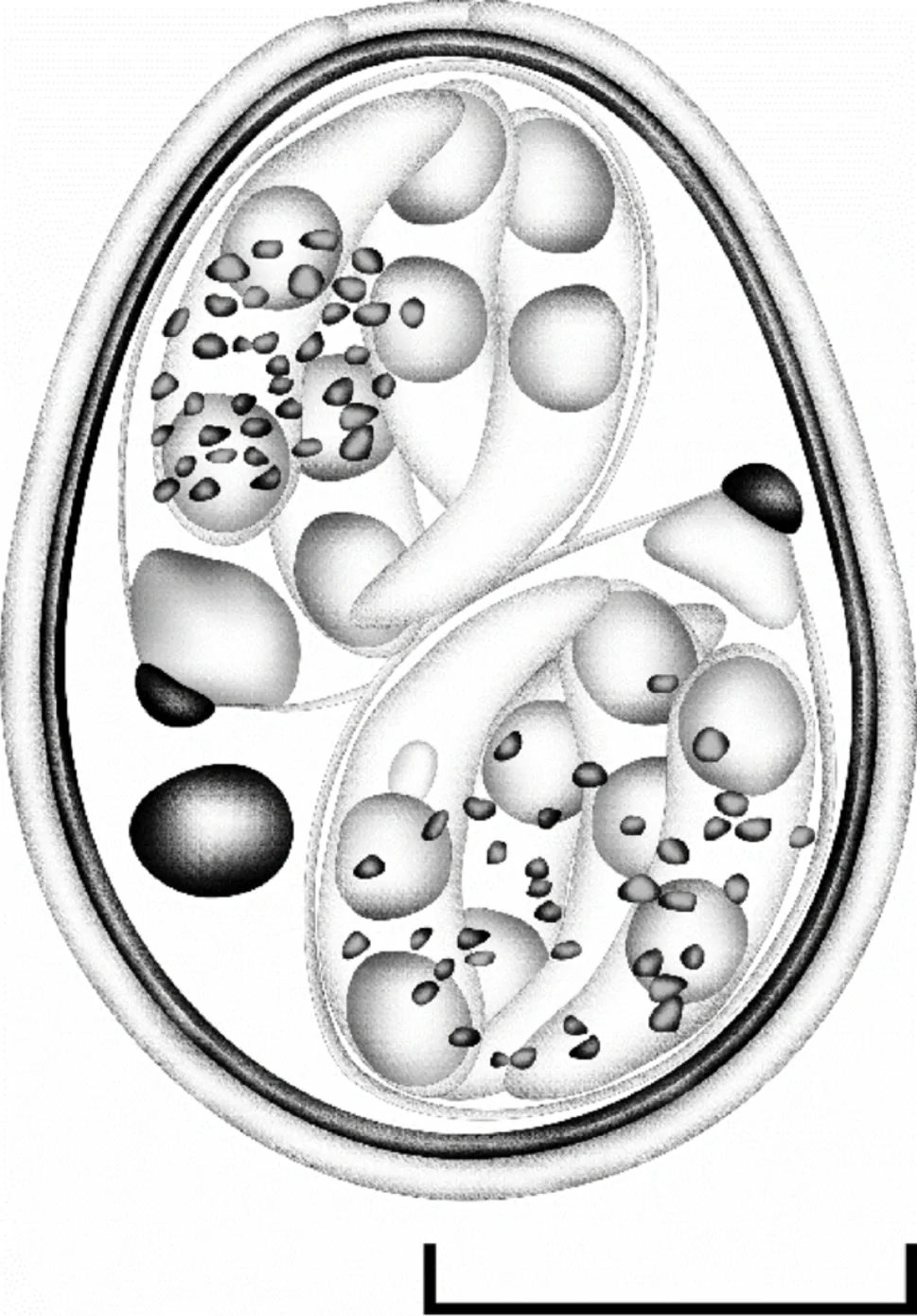
Composite line drawing of the sporulated oocyst of *Isospora atticorae* n. sp. from the White-thighed Swallow *Atticora tibialis* in Southeastern Brazil. Scale bar = 10 μm

**Fig. 2:**
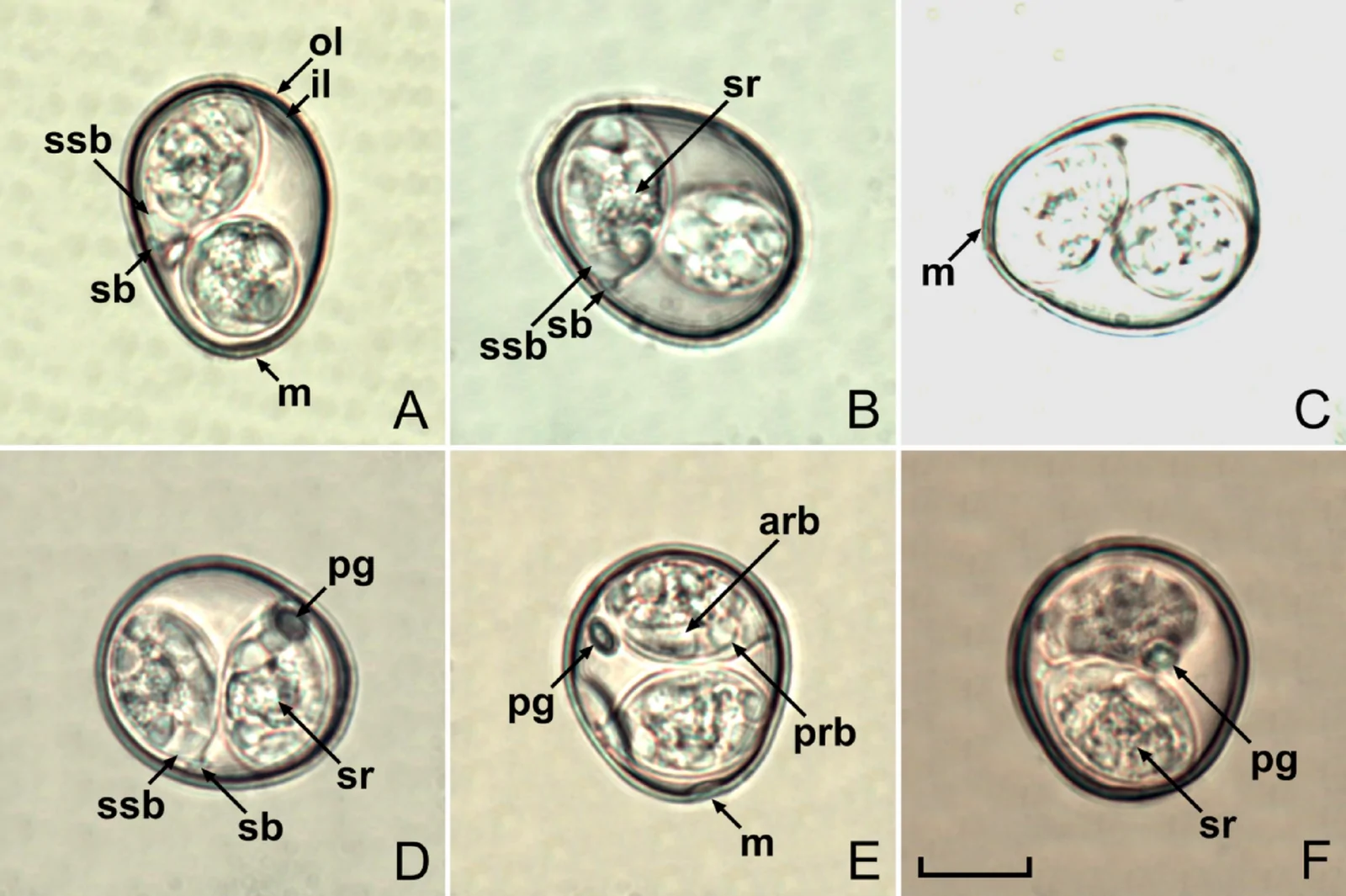
Photomicrographs of sporulated oocysts of *Isospora atticorae* n. sp. from the White-thighed Swallow *Atticora tibialis* in Southeastern Brazil. Anterior (arb) and posterior (prb) refractile bodies, outer (ol) and inner (il) layers of the oocyst wall, micropyle (m), polar granule (pg), sporocyst residuum (sr), Stieda body (sb) and sub-Stieda body (ssb) are indicated. All photomicrographs are shown at the same scale. Scale bar = 10 μm

*Isospora atticorae* Dorna-Santos et Berto n. sp. (Figures 1 and 2)

*Subclass*: Coccidia Leuckart, 1879.

*Family*: Eimeriidae Minchin, 1903.

*Genus*: *Isospora* Schneider, 1881.

*Sporulated oocyst* (*n* = 13): Oocyst shape pyriform (pear-shaped); 21–27 × 20–23 (24.4 × 21.2); length/width (L/W) ratio 1.0–1.3 (1.2). Wall bi-layered, 0.8–1.4 (1.1) thick; outer layer smooth, comprising approximately two-thirds of total thickness. Micropyle present, occasionally barely discernible but typically distinct, with or without an associated short invagination of the inner layer, 2.0–4.2 (2.9) wide. Oocyst residuum absent. Polar granule present, single and robust, 2.5–3.9 × 1.4–3.2 (3.3 × 2.2).

*Sporocyst and sporozoites* (*n* = 13): Sporocyst shape ellipsoidal to lemon-shaped; 14–18 × 9–11 (16.1 × 10.3); L/W ratio 1.4–1.7 (1.6). Stieda body present, knob-like, 0.7–1.4 × 1.4–1.9 (1.1 × 1.7). Sub-Stieda body present, rounded to trapezoidal, 1.5–2.5 × 2.6–3.7 (2.0 × 3.3). Para-Stieda body absent. Sporocyst residuum present, composed of granules totally scattered or loosely arranged among the sporozoites. Sporozoites vermiform, containing anterior and posterior refractile bodies and a barely discernible nucleus.

## Diagnosis

To date, four *Isospora* spp. have been described from hosts within the family Hirundinidae (Table 1). *Isospora atticorae* n. sp. can be distinguished from these congeners primarily by the presence of a micropyle (a feature unique to this species among hirundinid coccidians), in addition to other specific morphological and morphometric characteristics.

*Isospora hirundinis* Schwalbach, 1959, described from the Barn Swallow *Hirundo rustica* Linnaeus, 1758 in Germany, possesses larger, ellipsoidal oocysts compared to the smaller, pyriform oocysts of *I. atticorae* n. sp. (Table 1). Although Schwalbach [24] originally described the oocyst wall as single-layered, this likely represents a limitation of the microscopy at the time, as modern studies establish that a bi-layered wall is consistent across Eimeriidae coccidians [25–26]; this feature is clearly evidenced in the photomicrographs of the present study. Furthermore, *I. hirundinis* possesses pyriform sporocysts, whereas those of *I. atticorae* n. sp. are ellipsoidal to lemon-shaped. Based on the original line drawings, *I. hirundinis* appears to have a crescent-shaped (half-moon) Stieda body and a rounded sub-Stieda body, differing from the knob-like Stieda body and rounded-to-trapezoidal sub-Stieda body observed in the new species.

The comparison with *Isospora petrochelidon* Stabler et Kitzmiller, 1972, described from the Cliff Swallow *Petrochelidon pyrrhonota* Vieillot, 1817 in the USA, reveals marked structural differences (Table 1) [27]. *Isospora petrochelidon* exhibits two to three polar granules, whereas *I. atticorae* n. sp. consistently possesses a single, robust polar granule. Additionally, the sporocysts of *I. petrochelidon* are larger and more elongated than those of the new species. The Stieda and sub-Stieda bodies also differ significantly. While *I. petrochelidon* was originally described as having a Stieda body with a prominent cap, the original line drawings depict a flattened-to-knob-like structure, which remains distinct from the prominent, knob-like Stieda body of *I. atticorae* n. sp. Furthermore, the ellipsoidal sub-Stieda body of *I. petrochelidon* is easily distinguishable from the rounded-to-trapezoidal sub-Stieda body of *I. atticorae* n. sp. Finally, the sporocyst residuum in *I. petrochelidon* consists of a compact, rounded mass, contrasting with the dispersed granules observed in the new species.

*Isospora mehlhornii* Abd-Al-Aal, Ramadan et Al-Hoot, 2000 was also described from *H. rustica* in Egypt [28]. Regarding the oocysts, *I. mehlhornii* possesses larger, subspheroidal forms that lack a polar granule, whereas *I. atticorae* n. sp. has smaller, pyriform oocysts characterized by a prominent polar granule. In addition, the sporocysts of *I. mehlhornii* are larger than those observed in *I. atticorae* n. sp. (Table 1).

Finally, *Isospora zimmermani* McAllister et Hnida, 2023, recently described from *H. rustica*, shows some morphometric overlap with the new species (Table 1) [29]. However, *I. atticorae* n. sp. possesses pyriform oocysts, in contrast to the subspheroidal to ovoidal oocysts of *I. zimmermani*. Regarding the internal structures, *I. atticorae* n. sp. has a single polar granule, whereas *I. zimmermani* may possess up to three. Another distinct difference lies in the sporocyst residuum, which is a dense, compact sphere in *I. zimmermani*, while it remains dispersed among the sporozoites in *I. atticorae* n. sp.

## Taxonomic summary

*Type host*: *Atticora tibialis* (Cassin, 1853) (Aves: Passeriformes: Passeri: Passerida: Hirundinidae), White-thighed Swallow.

*Type locality*: Itatiaia National Park (22°26’S, 44°37’W), Southeastern Brazil.

*Type specimens*: Photosyntypes and line drawing are deposited in the Parasitology Collection of the LABICOC at UFRRJ, under repository number P-143/2026. This material is available online (http://r1.ufrrj.br/labicoc/colecao.html). Additionally, photographs of the type-host specimen (symbiotype) are deposited in the same collection.

*Representative DNA sequence*: DNA amplification of the cox1 gene (COIBF1 locus) yielded clear bands of approximately 250 bp. A representative sequence was deposited in the GenBank database under the accession number PZ445474.

*ZooBank registration*: To comply with the regulations set out in Article 8.5 of the amended 2012 version of the International Code of Zoological Nomenclature [30], details of the new species have been submitted to ZooBank. The Life Science Identifier (LSID) of the article is urn: lsid: zoobank.org: pub:6D346E03-3577-4F27-BD78-C18AF92DFCD5. The LSID for the new name *Isospora atticorae* Dorna-Santos et Berto n. sp. is urn: lsid: zoobank.org: act:68C37C60-6930–482 F-B37D-73D133AD0275.

*Site of infection*: Unknown; oocysts were recovered from feces.

*Prevalence*: 33.3% (1/3 hosts infected).

*Density*: An average of 7 oocysts per fecal droplet (based on two distinct droplets containing 6 and 7 oocysts, respectively).

*Etymology*: The specific name is derived from the generic name of the host, *Atticora*, declined in the genitive case (*atticorae*), following the rules of Latin grammar for the first declension (feminine).

### Molecular and phylogenetic analyses

The two DNA sequences obtained from the isolates of six and seven oocysts, respectively (all originating from distinct fecal droplets shed by a single *A. tibialis* specimen) were 100% identical. This sequence of *I. atticorae* n. sp. showed high similarity when compared with other *Isospora* spp. sequences deposited in the GenBank database. The highest identities, approximately 99% (with 2–3 nucleotide substitutions), were observed with *Isospora canaria* Box, 1975 reported from Island Canaries *Serinus canaria* (Linnaeus, 1758) in Brazil [31] and an unnamed *Isospora* sp. from American Robins *Turdus migratorius* Linnaeus, 1766 (unpublished data). *Isospora* spp. reported from oscine birds (Passeri) of different families, such as Mimidae, Estrildidae, Fringillidae, and Sturnidae, showed similarities of approximately 97–98%.

In contrast, *Isospora* spp. described from oscine and suboscine (Tyranni) birds in the Neotropical region (the same zoogeographical region as *I. atticorae* n. sp.) exhibited lower similarities (approximately 96–97%); these include *Isospora juruviarae* Andrade et Berto, 2024 from Chivi Vireos *Vireo chivi* (Vieillot, 1817) [32], *Isospora oliveirai* Ortúzar-Ferreira et Berto, 2020 from Greenish Schiffornises *Schiffornis virescens* (Lafresnaye, 1838) [33], *Isospora pichororei* Genovez-Oliveira et Berto, 2024 from Rufous-capped Spinetails *Synallaxis ruficapilla* Vieillot, 1819 [34], and *Isospora parnaitatiaiensis* Silva, Rodrigues, Lopes, Berto, Luz, Ferreira et Lopes, 2015 from White-shouldered Fire-eyes *Pyriglena leucoptera* (Vieillot, 1818) [35]. Beyond the genus *Isospora*, *Eimeria alorani* Hůrková, Baker, Jirků et Modrý, 2005 and *Eimeria apionodes* Pellérdy, 1954 from Striped Field Mice *Apodemus agrarius* (Pallas, 1771) were the non-congeneric species with the highest similarity, at approximately 95% [36].

**Fig. 3:**
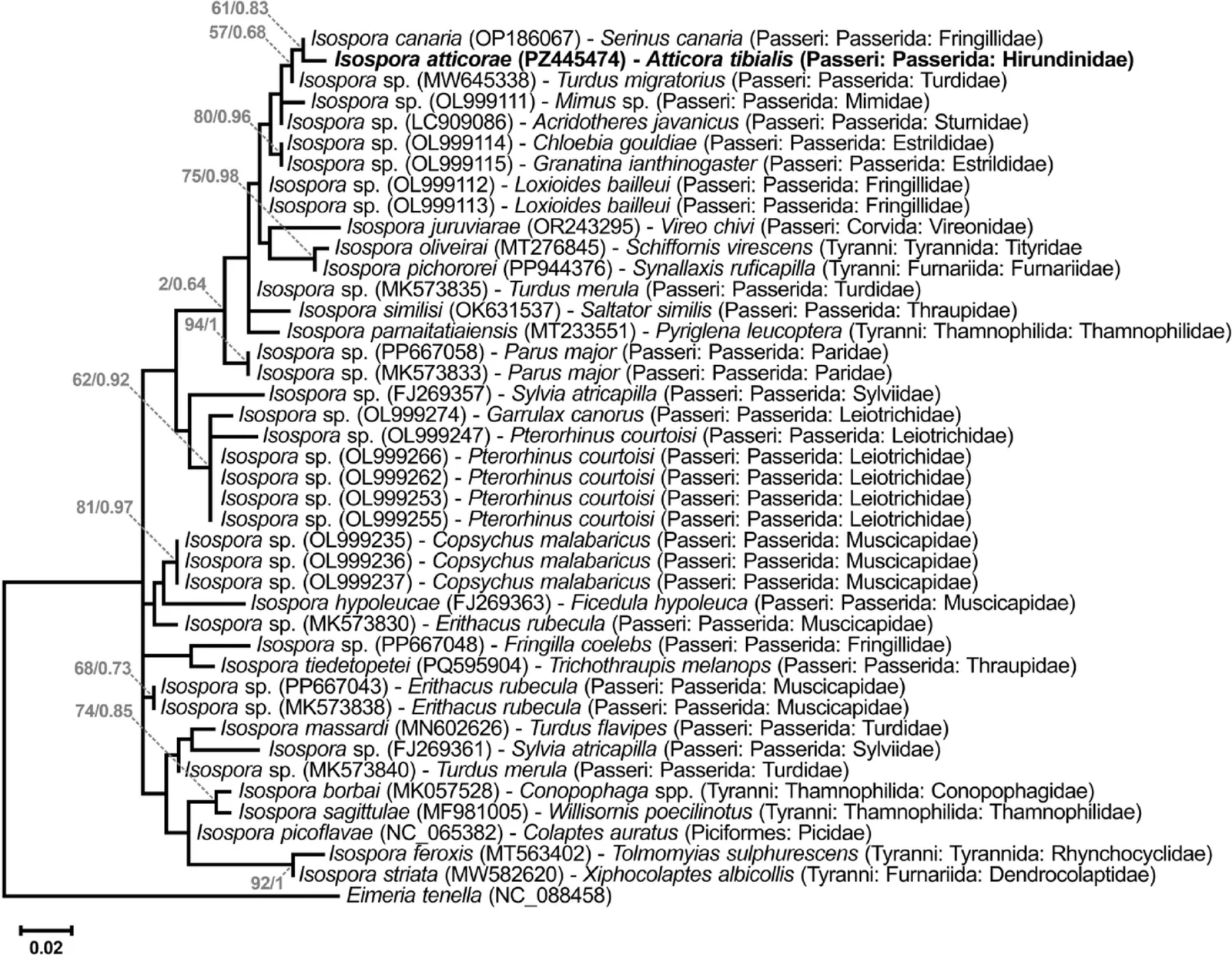
Phylogenetic relationships of *Isospora atticorae* n. sp. from the white-thighed swallow *Atticora tibialis*, inferred by Maximum Likelihood (ML) analysis of the mitochondrial cytochrome c oxidase subunit 1 (cox1) gene (257 bp). The tree is drawn to scale, with branch lengths representing the number of substitutions per site (scale bar = 0.02). Node labels indicate ML bootstrap support (left) and Bayesian posterior probabilities (right); only values > 50% (or 0.50) are shown. The phylogram was rooted with *Eimeria tenella* as the outgroup

The phylogenetic analysis based on the cox1 gene (Fig. 3) was congruent with the sequence comparisons, revealing well-supported monophyletic groups. *Isospora atticorae* n. sp. clustered as a sister group to *I. canaria*, and this clade subsequently grouped with the undescribed *Isospora* sp. from *T. migratorius*. Additionally, small, highly supported monophyletic clades comprising *Isospora* spp. from shared host species, families, parvorders, and/or suborders were observed throughout the topology. Among the species displaying higher genotypic similarity and close phylogenetic clustering within the same monophyletic group, only *I. canaria* could be morphologically compared. This is because the *Isospora* sp. from *T. migratorius* remains unpublished, and the operational taxonomic units corresponding to sequences OL999111–OL999115 and LC909086 lack sufficient morphological or morphometric description in the literature. *Isospora atticorae* n. sp. can be readily distinguished from *I. canaria* by possessing smaller, pyriform oocysts (24.4 × 21.2 vs. 27.2 × 24.7), the presence of a micropyle, and a distinct knob-like Stieda body. In contrast, *I. canaria* is characterized by its subspheroidal to ellipsoidal oocysts, the absence of a micropyle, and a typical half-moon-shaped Stieda body.

## Discussion

The family Hirundinidae currently comprises 21 recognized genera and 92 species [14, 37]. Despite their cosmopolitan distribution, migratory habits, and high conspicuousness in open areas globally where they forage in large flocks for aerial insects, the diversity of *Isospora* spp. described within this avian family remains remarkably underestimated [12, 29, 37]. This pronounced gap in the literature underscores the importance of taxometrical surveys targeting these hosts [12].

Hirundinids exhibit a widespread distribution in the New World, spanning from temperate North America to tropical and subtropical regions of South America [14]. Among the most extensively studied species, the cosmopolitan *H. rustica* stands out; its American distribution primarily encompasses North American breeding grounds, followed by seasonal migrations to Central and South America during the boreal winter [14, 37]. Similarly, *P. pyrrhonota* breeds in temperate North America and migrates to southern South America during non-reproductive periods [37]. Conversely, *A. tibialis* exhibits a strictly Neotropical distribution, restricted to the forested environments of the Amazon and Atlantic Forest biomes [3, 14].

Although geographic overlapping may occur between *H. rustica* and *A. tibialis*, these host species exhibit marked differences in habitat preferences [14, 37]. *Hirundo rustica* is strongly associated with open landscapes, rural pastures, agricultural zones, and heavily disturbed anthropogenic areas, frequently demonstrating synanthropic nesting habits by utilizing human constructions [10]. This high ecological plasticity significantly contributes to its colonization success and promotes high population connectivity across vast geographic regions [14, 37].

In contrast, *A. tibialis* is a predominantly resident species, strictly associated with humid forest environments, forest edges, riverbanks, and natural clearings [3, 10, 14, 37]. It relies on natural cavities and embankments for shelter and reproduction, displaying a negligible association with urbanized or intensely modified landscapes [37].

These biogeographical and ecological discrepancies carry major implications for the population dynamics and evolutionary dispersal of their associated parasites. The high vagility and migratory nature of *H. rustica* potentially favor continuous gene flow and connectivity among coccidian populations on a continental scale. Conversely, the relative ecological and geographic isolation of *A. tibialis* within complex forest biomes likely drives localized processes of parasitic diversification. This isolation, characterized by high environmental heterogeneity and natural barriers to host dispersal, strongly reinforces the morphological and molecular distinctiveness of *Isospora atticorae* n. sp. (Figures 1, 2 and 3), while supporting the hypothesis of narrow host specificity for this new coccidian species [38].

Beyond the natural dispersal and transmission dynamics dictated by the geographical ranges and ecological niches of their hosts, the potential role of wildlife trade (both legal and illegal) in the anthropogenic dispersion of coccidians warrants consideration [38]. The international wildlife trade represents one of the primary threats to global biodiversity, particularly impacting avian groups with high commercial, ornamental, or song value. Among the most severely affected families are Psittacidae, heavily targeted due to their vibrant plumage and mimicry capabilities, as well as Thraupidae, Cardinalidae, Fringillidae, and Turdidae, which are frequently exploited in the songbird trade [39–41]. This lucrative industry not only drives the population decline of trafficked species but also facilitates the anthropogenic dispersion of parasites and infectious agents among diverse avian hosts [38–39].

In stark contrast, the family Hirundinidae is historically absent from the major circuits of wildlife trafficking. Swallows garner negligible commercial interest when compared to traditional ornamental or songbirds, and they are virtually unrecorded in law enforcement seizures linked to the illegal trade [39–41]. Consequently, the geographical distribution patterns observed for *Isospora* spp. associated with hirundinids can scarcely be explained by anthropogenic factors [38]. This lack of human-mediated displacement reinforces why *I. atticorae* n. sp. possesses an exclusive combination of morphological traits, such as a distinct micropyle (Table 1), which evolved shaped by the natural history, migratory routes, and population isolation of its respective avian host. This contrasts sharply with coccidians infecting bird families like Thraupidae or Fringillidae, which are recurrently exposed to anthropogenic gene pool mixing due to the wildlife trade [38, 42].

Hirundinids possess predominantly insectivorous foraging habits, capturing their prey aerially during flight. Given that the transmission of *Isospora* spp. occurs strictly via the fecal-oral route, it can be inferred that ingested insects may act as mechanical vectors or paratenic hosts for these coccidians. This transmission hypothesis is supported by experimental evidence; for instance, Goodwin and Walman [43] demonstrated that beetles can mechanically carry viable, infective *Eimeria* oocysts and other pathogens adhered to their exoskeleton or within their digestive tract, successfully transmitting them to susceptible chicks. Furthermore, studies on the transmission dynamics of intestinal parasites indicate that various synanthropic and flying insects, such as dipterans and coleopterans, can mechanically transport cysts and oocysts [44]. Therefore, it is highly plausible that aerial insects exposed to environments contaminated with fecal droppings carry viable *Isospora* oocysts, thereby acting as efficient transmission agents among foraging swallows.

An alternative hypothesis that could be raised is that the oocysts observed in the feces of *A. tibialis* resulted from environmental contamination or pseudoparasitism, representing a passive passage through the intestinal tract due to the low number of positive hosts [45]. However, based on the criteria discussed by Andrade et al. [45], this hypothesis is highly improbable. Although oocysts were detected in only a single individual, the observed prevalence (33.3% out of three hosts) is well within the expected range for coccidians in free-living passerines [45–46]. Furthermore, the oocyst morphology aligns with the typical morphotypic patterns of other coccidian species described from Hirundinidae, supporting the taxonomic association of this morphotype with the host family. The pristine structural integrity of the oocyst walls and the turgid, bright appearance of the refractile bodies and sporozoites (Fig. 2) further weaken the pseudoparasitism hypothesis. Typically, oocysts undergoing purely passive passage through a non-specific digestive tract appear with wrinkled, altered, or partially ruptured walls, and with withered, opaque refractile bodies and sporozoites [45]. Methodologically, these oocysts were recovered from two distinct, isolated fecal droplets collected directly from clean paper liners at the bottom of the holding boxes, eliminating the possibility of external environmental contamination. Although examination of additional *A. tibialis* specimens would further corroborate this host-parasite association, this avian species is exceptionally rare to capture using mist nets; for instance, our research group has captured only three *A. tibialis* specimens over more than 12 years of continuous fieldwork in the PNI.

Another critical driver of *Isospora* infection in wild birds is sustained environmental exposure at nesting sites, particularly among avian species exhibiting colonial or semi-colonial breeding behaviors. Nests create a localized microenvironment highly conducive to coccidian transmission, as they provide warm, humid, and sheltered conditions enriched with fecal matter and organic debris. These abiotic factors optimize the sporulation rate and prolonged viability of oocysts, facilitating the infection of nestlings during parental feeding [4]. Additionally, the abundance of organic matter attracts diverse arthropods, which further amplifies transmission pathways via mechanical vectoring [43–44]. Consequently, the nest functions as a pivotal epidemiological unit where biotic and abiotic factors interact to maintain a continuous cycle of environmental contamination and early-stage infection [4].

Within this context, the detection of *I. atticorae* n. sp. oocysts in fecal samples of *A. tibialis* may reflect a chronic, long-term persistence of the parasite within the host population. In many passerines, early exposure in the nest leads to an extraintestinal cycle characterized as systemic isosporosis or avian atoxoplasmosis, where merozoites exploit mononuclear phagocytes for dissemination into visceral organs [4, 47–49]. Based on the biological models proposed by Box [47], such a systemic phase could theoretically provide a chronic tissue reservoir within host macrophages. Merozoites released from these extraintestinal sites back into the intestinal lumen would fuel continuous rounds of local replication, explaining how a resident, non-migratory host like *A. tibialis* could exhibit prolonged oocyst shedding even outside the breeding season.

However, it is important to emphasize that the existence of an extraintestinal cycle for *I. atticorae* n. sp. remains strictly speculative at this stage. Since our diagnosis relies exclusively on coprological analysis, the occurrence of systemic isosporosis in this host can only be proposed as a plausible hypothesis. Definite confirmation of an extraintestinal phase for this new species would mandate the direct identification of endogenous zoites within blood smears, mononuclear cells, or through histopathological and molecular evaluations of internal organs (e.g., liver, spleen, and lungs) [4, 47–49]. Future sampling incorporating these invasive diagnostic tools is required to elucidate whether *I. atticorae* n. sp. utilizes a purely intestinal development or shares the systemic, multi-organ lifecycles documented in other wild passerine coccidia.

To date, no extraintestinal *Isospora* sp. has been reported from hirundinid hosts; all named descriptions within this avian family have relied exclusively on the identification and morphology of oocysts recovered from fecal samples. *Hirundo rustica* has been the predominantly reported host for three of the four *Isospora* spp. described from hirundinids so far. This is highly correlated with its cosmopolitan distribution, migratory habits, and widespread occurrence in open landscapes and peri-urban areas [14, 37]. These ecological characteristics greatly facilitate its capture and sampling, resulting in a pronounced representational bias in the parasitological literature involving Hirundinidae.

Contrasting with this pattern, *I. petrochelidon* from *P. pyrrhonota* represents the only named species recorded from a different host [27]. In this framework, the identification of a new *Isospora* sp. from *A. tibialis* broadens the known host range associated with this coccidian genus, directly contributing to reducing the taxonomic and host-sampling bias currently observed in the literature.

Prior to this study, records of named *Isospora* spp. from New World swallows were strictly limited to the Nearctic, including *I. petrochelidon* and *I. zimmermani* (Table 1). However, both species were characterized solely based on morphological attributes [27, 29], lacking molecular data to support their taxonomic boundaries. Therefore, *I. atticorae* n. sp. represents the first species of the genus described from an integrative taxonomy perspective, simultaneously combining morphological and molecular data within the family Hirundinidae in the Neotropical region. This characterization substantially expands the knowledge regarding coccidian diversity and provides a pioneer integrative framework for parasites associated with Neotropical swallows.

Mitochondrial genes are highly suitable for species delimitation and comparisons among closely related taxa, whereas nuclear genes are generally preferred for resolving deeper phylogenetic relationships among broader taxonomic groups. This distinction arises because mitochondrial markers exhibit higher mutation rates over time and are less conserved than their nuclear counterparts [13, 50]. Given that the primary objective of the molecular analysis was a direct comparison with *Isospora* spp. sequences currently archived in GenBank, the mitochondrial cox1 gene was selected. Specifically, the locus COIBF1 targeted in this study is a pioneer marker in the molecular systematics of *Isospora* spp., originally established by Dolnik et al. [20]. This framework standardized the molecular identification of passerine coccidia and currently represents the marker for which the overwhelming majority of *Isospora* sequences are available in public databases.

The selection of *Eimeria tenella*, a non-congeneric species belonging to the same family (Eimeriidae), as an outgroup reinforces that the purpose of this analysis was not to reconstruct deep evolutionary relationships among higher-level taxa [13]. Instead, it aimed to perform precise species-level identification in comparison with available congeneric sequences.

Our phylogenetic analyses (Fig. 3) demonstrated that *I. atticorae* n. sp. forms a well-supported monophyletic group with other *Isospora* spp. associated with birds of the parvorder Passerida. This clade includes *I. canaria* from *S. canaria* in Brazil [31], as well as an unnamed *Isospora* sp. from *T. migratorius*. Although the avian hosts within the families Hirundinidae, Fringillidae, and Turdidae exhibit relatively distant phylogenetic relationships, the recovery of their respective *Isospora* spp. within a single monophyletic clade suggests that their common parasitic ancestor likely infected a basal ancestral passerid oscine bird, since Passerida represents the lowest common taxonomic rank sharing these three bird families [11, 38].

These interpretations align with a host-parasite cospeciation approach driven by narrow host specificity, a pattern also observable in other monophyletic groups containing *Isospora* spp. from Muscicapidae, Thamnophilidae, and Tyranni (Fig. 3). Admittedly, these clades are not perfectly congruent with all host families, meaning that parasites from each host family are not always uniquely bundled into exclusive, neatly delimited sister groups. Nonetheless, host-parasite cospeciation remains the most coherent evolutionary interpretation for the phylogenetic topology. This model stands in stark contrast to alternative hypotheses, such as common ancestry driven by geographical distribution (since *Isospora* spp. from identical type-localities clustered in distant and distinct clades) or oocyst morphology, as structural features appeared as homoplasious traits distributed heterogeneously across the phylogenetic tree. Although biological attributes, such as endogenous developmental stages and extraintestinal life cycles, could theoretically reveal alternative evolutionary signatures, the vast majority of these archived *Isospora* spp. currently lack such characterizations [13, 38, 51–52].

In this context, a relevant limitation of the present study is the scarcity of biological data and mitochondrial sequences for passerine coccidia deposited in public databases. Therefore, it is expected that a future expansion of both biological and molecular datasets will enable more robust evolutionary and phylogenetic inferences regarding the deep historical relationships between *Isospora* spp. and their avian hosts.

In conclusion, based on robust morphological and molecular evidence obtained from oocysts recovered from fecal samples of the White-thighed Swallow, *A. tibialis*, captured in the PNI, *I. atticorae* n. sp. is formally proposed herein as a new species. Consequently, *I. atticorae* n. sp. is established as the first representative from the family Hirundinidae globally to be characterized from an integrative perspective through the sequencing of a mitochondrial locus, as well as the fifth nominally described species in this avian family, and the first in the Neotropical region.

## Data Availability

No datasets were generated or analysed during the current study.
